# A ‘pocket guide’ to total internal reflection fluorescence

**DOI:** 10.1111/jmi.12070

**Published:** 2013-07-24

**Authors:** ML Martin-Fernandez, CJ Tynan, SED Webb

**Affiliations:** Central Laser Facility, Research Complex at Harwell, Rutherford Appleton LaboratoryHarwell Oxford, Didcot, UK

**Keywords:** Evanescent field, polarization, single-molecule fluorescence imaging, TIRF microscopy, total internal reflection

## Abstract

The phenomenon of total internal reflection fluorescence (TIRF) was placed in the context of optical microscopy by Daniel Axelrod over three decades ago. TIRF microscopy exploits the properties of an evanescent electromagnetic field to optically section sample regions in the close vicinity of the substrate where the field is induced. The first applications in cell biology targeted investigation of phenomena at the basolateral plasma membrane. The most notable application of TIRF is single-molecule experiments, which can provide information on fluctuation distributions and rare events, yielding novel insights on the mechanisms governing the molecular interactions that underpin many fundamental processes within the cell. This short review intends to provide a ‘one stop shop’ explanation of the electromagnetic theory behind the remarkable properties of the evanescent field, guide the reader through the principles behind building or choosing your own TIRF system and consider how the most popular applications of the method exploit the evanescent field properties.

## Plane wave disturbances at an interface – Snell's laws

**Fig 1:**
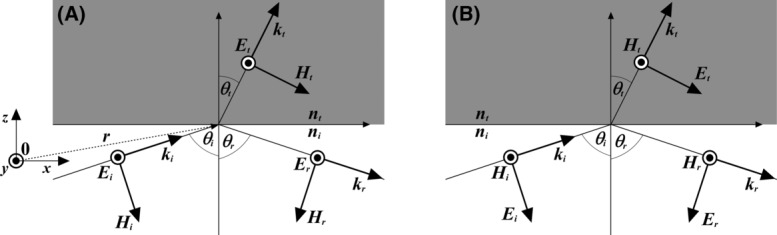
Plane waves at the boundary between two media. (A) *s*- and (B) *p*-polarized plane waves are polarized perpendicular and parallel to the plane of incidence, respectively. The electric (***E***) and magnetic (***H***) field components of the incident *i*, reflected *r* and transmitted *t* waves are orthogonal to each other and to the direction of propagation ***k***. From Snell's laws, *θ*_*i*_ = *θ*_*r*_ and *n*_*i*_ sin *θ*_*i*_ = *n*_*t*_ sin *θ*_*t*_.

Light is energy emitted by charged particles and manifests itself, as it travels through space, in the form of particles (photons) and electromagnetic waves. Electromagnetic waves have their oscillating electric (***E***) and magnetic (***H***) components perpendicular to each other and to the direction of propagation, defined by a wave vector *k* (Fig. 1). An electromagnetic wave at a position ***r***= (*x*, *y*, *z*), separated from its source by a distance much greater than its wavelength, is well approximated by a plane wave, described by infinite parallel planes of constant peak-to-peak amplitude normal to **k**. When an incident monochromatic plane wave described by an electric vector ***E****_i_* strikes the boundary between two optically transparent, isotropic media with refractive indices *n*_*i*_ and *n*_*t*_, there are reflected and transmitted monochromatic plane wave disturbances described by the electric vectors E_*r*_ and E_*t*_. These can be described as:(1.1)
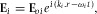
(1.2)
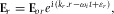
(1.3)
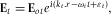
where ***E_o_*** is the maximum amplitude of the disturbance, *k*.*r* = *k*_*x*_*x* + *k*_*y*_*y* + *k*_*z*_*z* = *kr* cos *θ* is the dot product (or cosine projection) of ***k*** on ***r*** and *ε_r_* and *ε_t_* are phase constants relative to ***E****_i_* (as the position of the origin is not unique).

A number of field continuity conditions must be satisfied at the interface between two media. Specifically, the component of ***E*** tangential to the interface, which is shared by the disturbances on both sides, must be continuous across the boundary plane. From this condition, it is possible to derive Snell's laws, which describe the relationship between the angles of incidence *θ*_*i*_ and refraction *θ*_*t*_:(2.1)
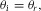
(2.2)
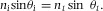


## Total internal reflection – the evanescent field

At an interface with a lower refractive index material (*n*_*i*_ > *n*_*i*_), light turns towards the boundary (Eq. (2.2.)). There is an incidence angle, known as the critical angle *θ*_*c*_ = sin^−1^ (*n*_*t*_/*n*_*i*_), at which *θ*_*t*_ has the largest possible value, which is *π*/2. At the critical angle, the refracted disturbance does not propagate through the medium with the lower reractive index, being ‘totally internally reflected’ at the interface. In these conditions, the expression describing the electromagnetic disturbance at the lower refraction index medium can be derived as follows: Let us make the incidence plane *y* = 0 and the interface *z* = 0. As **k**_*t*_ is in the incidence plane, it does not have a *y* component:(3)
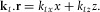
From Figure 1, using Snell's law (2.2) and the trigonometric formula (sin^2^ *θ*_*t*_ + cos^2^ *θ*_*t*_) we have:(4)
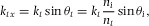
(5)
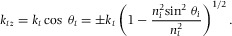
For totally internally reflected light, sin *θ*_*t*_ > sin *θ*_*c*_ = *n*_*t*_/*n*_*i*_. This makes the radicand in (5) negative. Given that 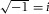, the square root is an imaginary term and we have:(6)
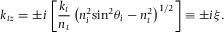
Substituting (7) and (6) in (1.3), the expression describing the electromagnetic disturbance in the lower refraction index medium is:(7)
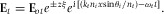
As a positive exponential diverging to infinity is physically unsustainable, the wave amplitude is **E**_*ot*_*e*^−*zξ*^. Equation (7) shows, therefore, that when *θ*_*i*_ > *θ*_*c*_ the electromagnetic field decays exponentially as it penetrates deeper into the media with the smaller refraction index. A near-field ‘evanescent’ wave therefore forms at the boundary, which propagates along the surface in the *x*-direction.

**Fig 2:**
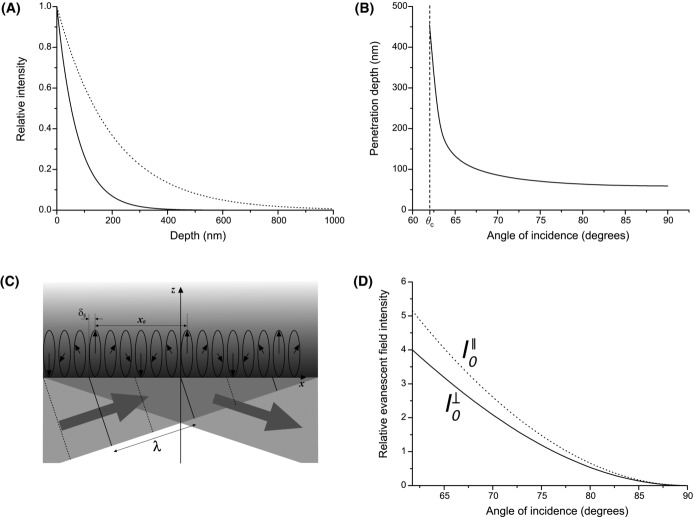
The evanescent field. (A) Profile of the evanescent field for the minimum (solid line) and maximum (dotted line) possible angles of incidence in a typical TIRF microscope, incorporating an objective with a numerical aperture of 1.45, and where the lens, immersion oil and cover slip glass have a refractive index of 1.51 and the sample has the same refractive index as water (1.33), *λ* = 532 nm. In practice, the incident beam has a finite width and hence a (small) range of incidence angles. Note that, the dotted line represents the evanescent field at the critical angle. (B) The evanescent field depth reduces as the angle of incidence increases. The optimum angle will depend on the proximity of the sample plane to the interface at which total internal reflection occurs and on the thickness of optical section required. (c) For an incident wave polarized in the *x*–*z* plane (*p*-polarization) undergoing total internal reflection, the polarization direction of the evanescent field cartwheels along the *x*-axis with period *x*_0_. Note that, the polarization of the evanescent field is shifted with respect to the incident wavefronts by *δ*_||_, this is the Goos–Hänchen shift. Only the wavefronts of the incident wave are shown. (d) The intensity of the evanescent field can be five times greater than the incident illumination beam, when the angle of incidence is close to the critical angle.

The intensity *I* of an electromagnetic field refers to the power per unit area that the field dissipates at the boundary and is proportional to 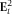. From Eqs. (6) and (7), the intensity of the evanescent field also exponentially decays along the *z-*direction perpendicular from the interface:(8)
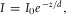
where *d* is the depth of penetration of the evanescence field. From Eq. (6), *d* is defined as:(9)
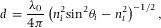
where *λ*_0_ is the wavelength of light in vacuum. Being dependent only on *θ*_*i*_, *n*_*i*_ and *n*_*t*_, the depth of penetration is independent of the polarization of the incident light. Equation (9) shows that *d* decreases as *θ*_*i*_ increases and is comparable to *λ*_0_ or smaller (except for *θ*_*i*_ = *θ*_*c*_, when *d* → ∞). Figure 2 shows the profile of the evanescent field for the maximum and minimum angles of incidence possible in a typical total internal reflection fluorescence (TIRF) microscope, although Figure 2(B) shows the variation in evanescent field depth as a function of the angle of incidence.

## Polarization properties of the evanescent field

To fully describe the evanescent wave (E_*t*_ = *E*_*e*_) we need to know the form of the electric vector E_*oe*_ = (*E*_*ox*_, *E*_*oy*_, *E*_*oz*_) at the boundary. This can be written as a function of its components parallel and perpendicular to the incidence plane (Fig. 1):(10)
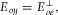
(10.2)
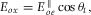
(10.3)
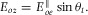
However, expressions that are useful are those that express these components as a function of 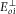, 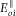 and *θ*_*i*_ (Fig. 1), which are the parameters we can experimentally control, and Fresnel's equations (Box 1) and Snell's laws allow us to do this. Defining 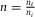 and using the following identities: 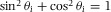, 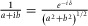 (with 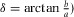
and *i* = *e*^*i*(*π*/2)^, they allow us to write:(11)
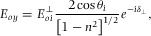
(11.2)
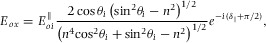
(11.3)
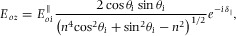
where *δ*_⊥_ and *δ*_||_ are phase factors given by:(12.1)
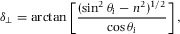
(12.2)
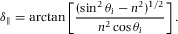


Note that, the direction of the evanescent field is perpendicular to the *x-*direction of propagation only when the incident electromagnetic disturbance E_*oi*_ is ⊥ to the incidence plane, also known as *s*-polarization (as E_*oe*_ = *E*_*oy*_). By contrast, the evanescent field amplitude E_*oe*_ = *E*_*ox*_ + *E*_*oz*_ is elliptically polarized in the incidence plane when *E*_*oi*_ is ∥ to the incidence plane, also known as *p*-polarization, and ‘cartwheels’ along the boundary, as the wave propagates in the *x*-direction, with a spatial period *x*_0_ (Fig. 2(c)). The value of *x*_0_ is derived from the imaginary exponent in Eq. (7) from:(13)
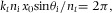
from which *x*_0_ = *λ*_0_/(*n*_*i*_ sin *θ*_*i*_).

Another interesting property is the presence of the phase factors *δ*_⊥_ and *δ*_||_, which give rise to a small but measurable longitudinal shift known as the Goos–Hänchen shift (Axelrod *et al.*, 1984). This shift reflects the distance which light moves along the surface within the lower refractive index material before being totally reflected back into the higher refractive index material.

From Eq. (11), the evanescent field intensity at the boundary as a function of the incident field is:(14)
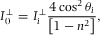
(15)
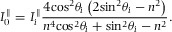


The evanescent field can therefore be several times stronger than the intensity of the incident light for incidence angles within a few degrees of the critical angle (Fig. 2(d)). Note that, the intensities of both components are different, with 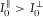.

**Box 1. Fresnel's equations**

An electric vector E_*i*_ can always be resolved into components parallel and perpendicular to the incidence plane. If E_*i*_ is perpendicular to the plane of incidence (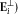, as in Figure 1(A), then the magnetic vector H_*i*_ is parallel to it. The tangential component of **H** must be continuous at the boundary and therefore,
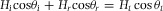

From electromagnetic theory, H = (*μ*/*ν*) E. In dielectric isotropic media (e.g. water, glass, immersion oil, etc.), the magnetic permeability *μ* = 1. Since *θ*_*i*_ = *θ*_*r*_ and *n* = *c*/*v*, we can write:
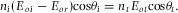

Using Snell's laws, we then have one pair of Fresnel's equations:
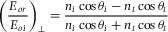
and
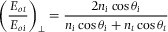

Similarly, if E_*i*_ is parallel to the plane of incidence (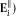, as in Figure 1(B), the continuity of the tangential component across the boundary leads to:
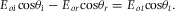

From the continuity of the tangential components of ***H***, we have:
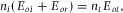
and therefore the other pair of Fresnel's equations are:
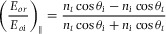
and
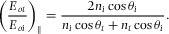

## Setting up a TIRF microscope

A TIRF microscope is designed to induce an evanescent field at the interface of two transparent isotropic media with different refractive indices close to or within a microscope sample. Most systems use laser beams as incident light. In cell biology applications, the interface at which the evanescent field is created is usually between the glass of a cover slip (*n*_*i*_ = 1.51) and a film of aqueous (buffer) solution between the cover slip and cells adherent to it (*n*_*t*_ = 1.33). There are two main approaches to creating an evanescent field: one is prism-based and the other objective-based.

**Fig 3:**
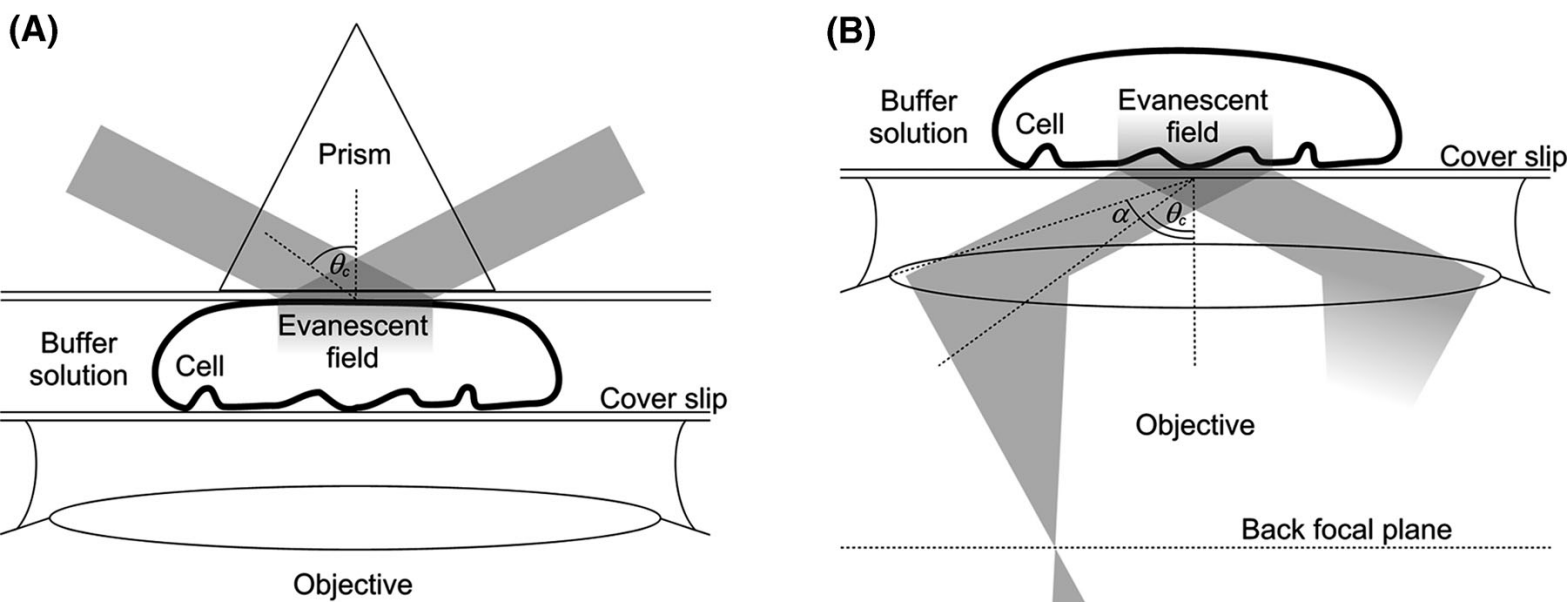
Prism- and objective-type TIRF. (A) In prism-type TIRF microscopy, the evanescent field is created on the opposite side of the sample from the objective lens that collects the fluorescence. The incident angle may have any value between the critical angle *θ_c_* and π/2. (B) With objective-type TIRF microscopy, fluorescence is collected from the sample on the same side as the excitation light is delivered. The range of incident angles is limited, as described by the numerical aperture of the objective, to angles between *θ_c_* and *α*. A collimated excitation beam is achieved by focusing it off-axis in the back focal plane.

In prism-based TIRF microscopy, a prism is attached to the surface of the cover slip, which directs the laser light towards the cover slip/buffer interface (Fig. 3(A)). Note that, if the specimen is a monolayer of adherent cells in culture, the use of a prism requires an upright fluorescence microscope with a water immersion objective lens. The main advantages of prism-based TIRF are that the incident angle can be large so that the evanescent field is consequently thin and that the unwanted reflected disturbance is not collected by the objective together with the excited fluorescence from the sample. The major disadvantage is that the fluorescence excited by the evanescent field is generated on the opposite side of the sample to the objective lens, resulting in the need to focus through the specimen. The increased refraction across the sample also leads to levels of spherical aberration difficult to correct for once the distance to the objective exceeds a few tens of micrometres. Because of this, prism-based TIRF is not as suitable for thicker cell types, such as some of mammalian origin, or tissue sections. In addition, setting up procedures associated with adding/removing the prism and realigning the beam may be required each time a new sample is used, making the method more cumbersome than objective-based TIRF.

Currently, most TIRF microscopes are objective-based. In these, light is delivered to the sample and collected from it by the objective lens, a method that requires the use of an inverted fluorescence microscope (Fig. 3(B)). Light reaches the objective via the microscope excitation path which can include a commercial TIRF slider or custom-made TIRF optics. Compared with the prism method, the objective-based method is more convenient as the specimen is easily accessible and the angle of incidence of the laser light can be changed easily. A disadvantage is that, as well as the fluorescence from the sample, the specular reflection of the incident beam is also efficiently collected by the objective. With weakly fluorescent samples, efficient notch filters must be used to prevent the reflected beam from hitting the detector. It is also important to use suitable dichroic beam splitters to minimize interference fringes in the illumination pattern; modified set-ups have been devised to remove them entirely (Mattheyses *et al.*, 2006). In custom systems, this specular reflection can be used to verify that TIRF has been achieved (Vizcay-Barrena *et al.*, 2011).

To achieve total internal reflection, the incident light must emerge from the objective at an angle *θ*_*i*_ from the optical axis larger than the critical incidence angle *θ*_*c*_ for the cover slip/buffer interface. From Eq. (2.2), this critical angle is *θ*_*c*_ = arcsin(*n*_water_/*n*_glass_) = 61°. Total internal reflection also requires that the light emerges as a parallel beam (i.e. a train of electromagnetic plane waves); otherwise, one would only achieve partial internal reflection for those rays with *θ*_*i*_ > *θ*_*c*_. To achieve this, the laser light must be focussed at the objective's back focal plane (Fig. 3(B)). When light is focussed in the middle of the back focal plane, the beam exiting the objective will be parallel to the optical axis (*θ*_*i*_ = 0); this is comparable to standard epi-illumination. By translating the focus beam away from the optical axis along the back focal plane one can increase the value of *θ*_*i*_. The maximum value of *θ*_*i*_ that can be achieved is determined by the numerical aperture (NA) of the objective. This is a number that characterizes the range of angles over, which the lens can emit or accept light, and is defined as:(16)
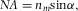
where *n*_*m*_ is the refractive index of the medium the objective is immersed in, and *α* is the field aperture, i.e. the maximum half-angle of the light cone accepted by the objective lens from the sample (Fig. 3(B)). For a parallel beam emerging from the objective, *θ*_*i*_ can equal α.

Given that sin*α* ≤ 1, TIRF is theoretically possible at the cover slip/buffer interface if the objective lens has an *NA* > *n*_water_ = 1.33. In practise, an *NA* ≥ 1.45 is required for three reasons. First, it is extremely difficult to obtain a laser spot small enough to achieve a good parallel beam out of the objective with an *NA* < 1.45. Tighter focusing typically requires the additional turning of light and increased angle of the light collection cone provided by very high NA objectives. The second reason is that, in cell biology experiments, the interface at which the evanescent field is induced is not a continuous surface, but rather a stratified multilayer system of refractive indexes varying between 1.33 (water) and ∼1.38, which is the refractive index of the primary cellular component (cytosol) in contact with the glass. Finally, the larger the NA of the objective, the larger the percentage of the peripheral area of the lens that can be utilized for total internal reflection, making coupling of the laser into the rear aperture less challenging.

To reach an *NA* ≥ 1.45, the objective must be immersed in a medium with *n*_*m*_ > 1.45. Oil immersion is used because its refractive index (*n*_oil_ ≃ 1.51) matches that of glass, reducing unwanted reflections. Note that, the higher the NA, the lower the possible penetration depth of the evanescent field. The optimal NA may therefore depend on the separation expected between the molecules to be excited by the evanescent field and the interface, which may vary as in molecules at the plasma membrane of cells that show ruffles and invaginations (Fig. 3(B)). It should also be noted that in multicolour applications, the different laser wavelengths that have to be combined will produce different penetration depths (Eq. (9)). In these cases an apochromatic objective and chromatically corrected optics throughout should be used.

## Applications of TIRF microscopy

The applications of TIRF microscopy follow from the specific properties of the evanescent field, which are not shared by other fluorescence microscopies. Many applications make use of several of these properties, but it is helpful to identify which is key in each case.

### The evanescent field intensity decreases exponentially with distance from the interface

**Fig 4:**
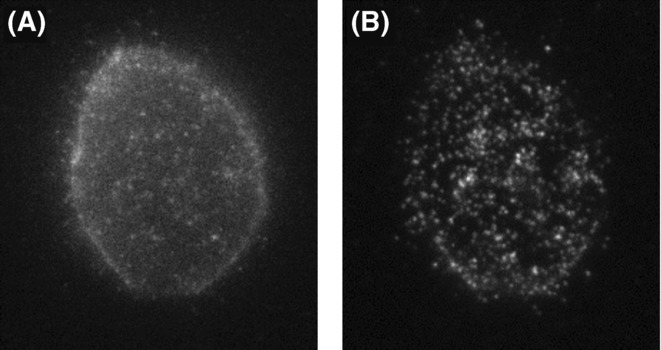
Comparison of epifluorescence and TIRF imaging. (A) A CHO cell with epidermal growth factor (EGF) labelled with CF640R and excited under (A) epi-illumination and (B) TIR-illumination. In the TIRF image, we see only those EGF molecules bound to receptors in the outer cell membrane closest to the cover slip. Each spot is the fluorescence from one (or another small integer value) CF640R molecule. Compared to the epi-illumination image, the enhanced intensity from these molecules and the reduced intensity from elsewhere in the cell mean that the signal-to-background ratio is much greater and many more individual molecules can be detected.

(Eq. (8)). The limited penetration depth of the evanescent field can be exploited to achieve selective excitation of the sample in the immediate vicinity of the glass/buffer interface – approximately within *d* < 200nm from the boundary (Eq. (10)). In mammalian cells, whose depth is typically a few micrometres, only a small section at the base of the cell is illuminated by the evanescent field. Naturally occurring fluorophores in the cell bulk, such as NADH and flavins, are therefore not significantly excited by the evanescent field and very high signal-to-background ratios (SBR) can be achieved. This is clearly seen in Figure 4, where the same cell is seen under both epi and TIR illumination. This form of ‘optical sectioning’ has proved ideal for imaging fluorescence from cellular features at the cell/substrate interface, such as focal adhesions, secretory vesicles, early endocytic particles, etc. (Axelrod, 2001). The shape of the exponential decay has also been used to obtain information on the axial position of the features of interest. For example, by ratioing the intensity of fluorescent chathrin-coated pits in epi- and TIRF-illumination, one can follow the axial positions of these vesicles during internalization with 10 nm resolution (Saffarian & Kirchhausen, 2008).

Although the interface at which total internal reflection occurs is usually at the cover slip, the different refractive indices within a sample may also permit the interface to be located deeper in the sample. This has allowed imaging of the plasma membrane in plant cells, which, until recently, was considered impossible because the cell wall is thicker than the evanescent field (Vizcay-Barrena *et al.*, 2011). Note that, depending on the refractive indices present in a sample, ‘frustrated TIRF’ may also occur, in which the field created at the cover slip interface generates propagating light at a second interface located within the evanescent field depth (Wan *et al.*, 2011). Frustrated TIRF is readily distinguished because the beam reflected from the interface is much weaker.

As noted above, a direct consequence of the exponential decay of the evanescent field is that the bulk of thick samples is not illuminated. This makes TIRF microscopy ideal for the long-term imaging of cells, in which the normal function of its organelles may be perturbed by even moderate photon fluxes (Frigault *et al.*, 2009).

## The evanescent field intensity is greater than the incident intensity

(Eqs. 14 and 15). As already noted, the intensity is boosted for incident beam angles within about 10° of the critical angle, improving the signal-to-noise ratio (SNR). The combination of high SNR and high SBR in TIRF microscopy has facilitated the development of a variety of methods that depend on single-molecule detection on the basolateral surface of cells (Selvin & Ha, 2007). Although TIRF illumination is not always required for single-molecule sensitivity, its characteristics mean that it is used where possible. In Figure 4(B), it is clear that TIR illumination allows more single molecules to be detected and that they are brighter than in the corresponding epi-illuminated image.

These include single-molecule tracking, which aims to determine the patterns of interaction between molecular species and the kinetic rates of association and dissociation from the position of interacting molecules versus time (good examples in Hern *et al.*, 2010); single-pair fluorescence resonance energy transfer (FRET), which uses Van der Waals nonradiative interactions between the two molecular dipoles of an energy donor and an energy acceptor (which depend as *R*^−6^, where *R* is the distance between the molecules) to characterize short range (<8 nm) intermolecular interactions and intramolecular conformational changes (Roy *et al.*, 2008); and fluorophore localization-based super-resolution techniques, such as stochastic optical reconstruction microscopy (van de Linde *et al.*, 2011).

## The evanescent field can have polarization components in each of the three spatial axes

(Eq. (11)). The existence of a polarization component along the optical axis of the microscope (i.e. the *z*-axis) is unique to TIRF microscopy – with epi-illumination, the field propagates along this axis and only has components in the *x*–*y* plane. When the incident light is *p*-polarised (in the *x*–*z* plane), the *x-*component is much smaller (about 5%) than the *z-*component and so E_*oe*_ ≃ *E*_*oz*_. The corresponding *s*-polarization is parallel with the *y*-axis. (Alternatively, *p*-polarization can be in the *y*–*z* plane and *s*-polarization parallel with the *x*-axis.) Given that the probability of photon absorption depends on the dot product of the fluorophore's excitation dipole and the polarization of the excitation field, the fluorescence intensity from the molecule must also be dependent on its orientation and the evanescent field polarization. By switching the polarization of the incident light between the *s*- and *p*-directions to alternate the evanescent field polarization between the *y*- and *z-*directions and recording for each the fluorescence intensity from the molecule, one can determine the orientation of its excitation dipole (Forkey *et al.*, 2000).

In conjunction with dyes that align orthogonally to the membrane, this has been used to measure the local orientation of cell membranes to show ruffles and invaginations. Provided that the fluorophore's excitation and emission dipoles are aligned, i.e. the fluorescence anisotropy in solution is close to 0.4, then the orientation of the molecule may be quantified by resolving the *x* and *y* components of its emission. When performed at the single-molecule level, this enables the three-dimensional orientation of individual macromolecules immobilized on glass to be measured quantitatively to derive structure-function relationships. Suitable fluorophores include (rhodamine, fluorescein, Cy3, etc., Shi *et al.*, 2012). Note that, regardless of how a molecule is excited, the polarization of the fluorescence from a single molecule is determined solely by the orientation of its emission dipole and so FRET and orientation measurements may be combined (Webb *et al.*, 2008). By contrast, the emission polarization in ensemble experiments depends on the excitation polarization via photoselection, the presence of FRET leading to depolarization and the loss of orientation information.
